# Digital infrared thermal imaging of udder skin surface temperature: a novel non-invasive technology to monitor calving process in Murrah buffalo *(Bubalus bubalis)*

**DOI:** 10.1038/s41598-023-40447-4

**Published:** 2023-08-14

**Authors:** Allu Teja, Jeyakumar Sakthivel, K. Ananda Rao, Arumugam Kumaresan, K. P. Ramesha, Narayanan Krishnaswamy, C. Gowtham Varma, M. Sivaram, Maharajan Lavanya, Vedamurthy Gowdar Veerappa, Mukund A. Kataktalware, D. N. Das, Kaushik Majumder, Niribili Rajbangshi

**Affiliations:** 1 Veterinary Gynaecology and Obstetrics, Livestock Research Centre, Southern Regional Station of ICAR-National Dairy Research Institute, Adugodi, Bengaluru, 560030 India; 2 Buffalo Research Station, Sri Venkateswara Veterinary University, Venkataramannagudem, 534101 Andhra Pradesh India; 3 Dairy Production Section, Southern Regional Station of ICAR-National Dairy Research Institute, Adugodi, Bengaluru, 560030 India; 4 Veterinary Gynaecology and Obstetrics, Theriogenology Lab, Southern Regional Station of ICAR-National Dairy Research Institute, Adugodi, Bengaluru, 560030 India; 5 ICAR-Indian Veterinary Research Institute, Hebbal Campus, Bengaluru, 560024 Karnataka India; 6 Veterinary Assistant Surgeon, Department of Animal Husbandry, S.V. Veterinary University, Tirupati, DAH, Government of Andhra Pradesh, Vijayawada, India; 7 Dairy Economics and Statistics Section, Southern Regional Station of ICAR-National Dairy Research Institute, Adugodi, Bengaluru, 560030 India

**Keywords:** Biophysics, Health care, Medical research

## Abstract

Quantifiable decline in the maternal body temperature during the pre-calving offers the possibilities for predicting the calving that can improve the calving management. As infrared thermography (IRT) is a simple non-contact tool for precise measurement of surface temperature, we investigated the use of IRT to establish thermal signatures around calving in the Murrah buffalo. The IRT of eye, right lateral, left lateral and rear side of udder skin surface temperature (USST) were recorded at 6 h interval from 96 h before the expected date of calving, at the time of calving and 24 h post-calving in Murrah buffaloes (n = 28). In parallel, blood samples were collected for progesterone (P_4_) assay. The results revealed that the IRT of the eye, right and left lateral and rear side of USST showed a significant decrease in the temperature from 48 h pre-calving till the onset of calving with a ΔT (°C) of 0.56, 0.91, 0.70, and 0.90, respectively. Mean USST significantly declined from 48 h pre-calving with a ΔT of 0.85 °C. The residual temperature of both eye and various ROI of the udder also followed a similar and significant declining trend from 48 to 0 h of calving indicating that circadian influence on the USST was minimum. Plasma P_4_ concentration significantly decreased from 72 h pre-calving till calving. It is concluded that a marked reduction in the IRT of the USST at 6–12 h pre-calving would be useful in predicting the onset of calving in the Murrah buffalo.

## Introduction

India is native to 20 breeds of buffaloes^1^ that contribute 45.07% of the annual milk production of 221.06 million tons^2^. Dystocia is a key concern for the buffalo dairy farmers as it adversely affects the viability of dam and calf, productive and reproductive potential of the dam^3^. Like cow, most calvings occur during the odd hours of night or wee hours of the day in the buffalo^4^. As assistance should be given within 65 min of the appearance of the fetal part(s) to maximise the survivability of both dam and calf in Holstein cows^5^, the impending calver should be shifted to a separate calving pen. Therefore, an accurate calving time prediction is important to provide timely assistance to save the life of dam and new born calf, welfare and to enhance the overall herd profitability^6^.

Devices like accelerometer or inclinometer, pressure sensors, temperature sensors^7,8^ and hormonal changes^9,10^ are used to predict calving. Thermal sensors are potential predictors of calving as body temperature appreciably decreases within 72 h pre-calving^6^. Though many invasive and semi-invasive automated body temperature monitoring technologies are available^11–18^, thermal profiling with digital infrared thermography (IRT) has advantages over the existing methods and is used in the cow^19^. The IRT profile of eye and vulval temperature around calving showed a sharp reduction of temperature at 12 h prior to onset of calving with a temperature difference (ΔT) of 0.54 and 0.39; 0.39 and 0.32 °C in Deoni (*Bos indicus*) and Holstein Friesian crossbred (*Bos indicus* x *Bos taurus*) cows, respectively^20^.

Visual cues such as udder development, tumescence of teats, bagging up of udder, vulvar edema indicate impending calving in a buffalo^10^. Udder temperature is closely correlated with body temperature in non-mastitis cows^21,22^. The IRT is sensitive enough to detect the changes in udder skin surface temperature (USST) caused by mastitis, milking, environmental temperature, and exercise^23^. The thermal window of udder region covers the body of mammary tissue to capture the radiation emitted by the mammary gland’s cutaneous artery and veins. We hypothesized that the IRT profile of eye and USST would be predict the onset of calving time in the Murrah buffalo.

## Results

Macro and micro weather parameters recorded inside the calving pen during the study period (August 2021 to February 2022) is given in Supplementary Table S1. Temperature humidity index (THI) influence during the study period did not reveal any effect on the skin surface temperature in the Murrah buffaloes indicating lack of seasonal effect. Exploratory data analysis of the IR temperature from the different regions of the interest (ROI) within 96 h of calving indicated that the data after 24 h post-calving did not vary. Descriptive statistics on the IR temperature of the eye, USST of right lateral, left lateral, rear side and overall mean of all three sides of udder prior to 96 h, 0 h calving and 24 h post calving is presented in Supplementary Tables S2.1, S2.2, S2.3, S2.4 and S2.5.

**Figure 1:**
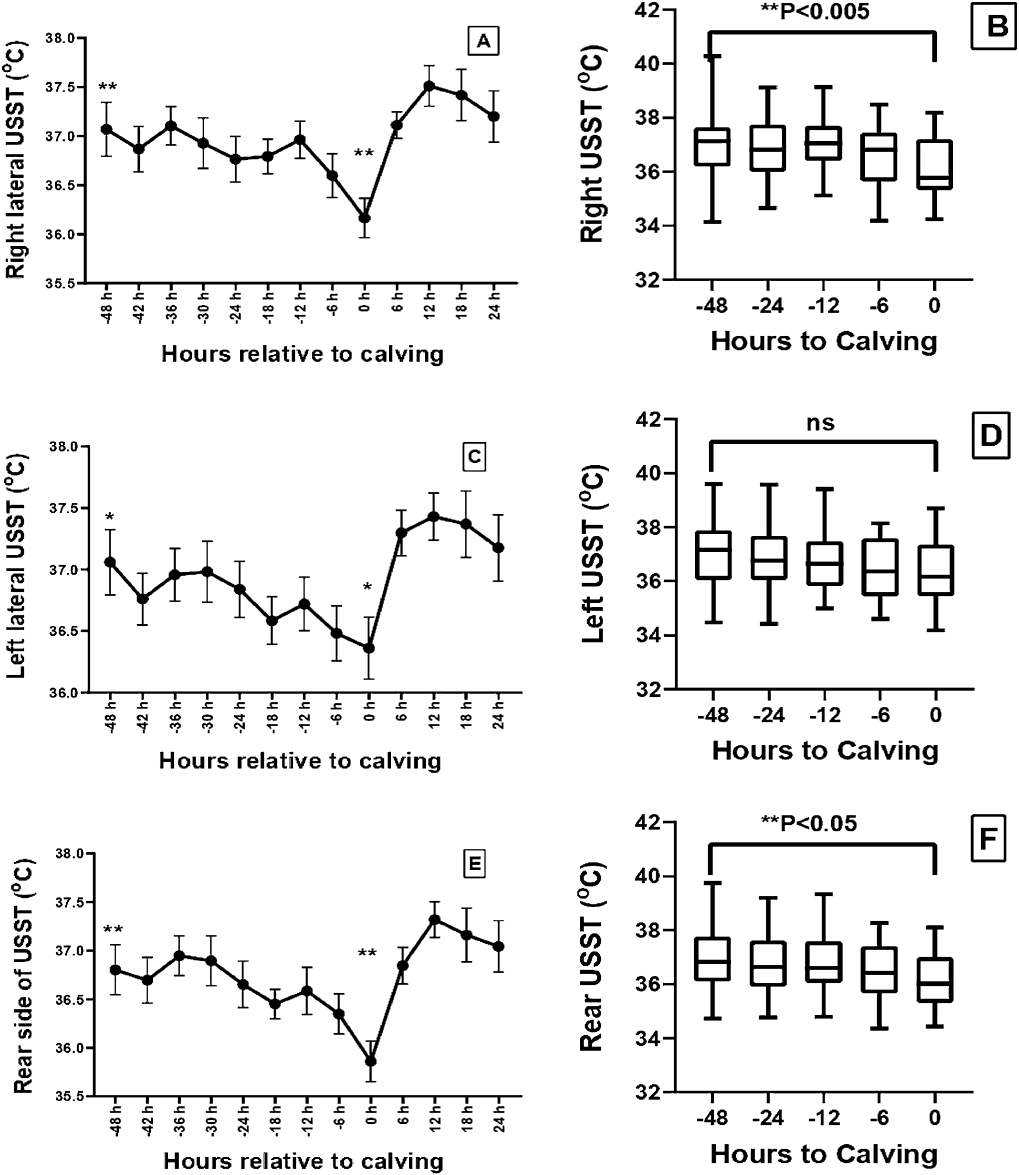
Infrared thermal profile of udder from 48 h prior to calving to 24 h post calving in buffaloes. (**A**) Right lateral udder skin surface temperature, (**B**) distribution of Tmax of right lateral udder skin surface; (**C**) left lateral udder skin surface temperature, (**D**) distribution of Tmax of left lateral udder skin surface; (**E**) Rear side udder skin surface temperature, (**F**) Distribution of Tmax of rear side udder skin surface. Asterisks (**) indicate the time points with significant difference in mean surface temperatures at P < 0.05. Values presented as mean ± SE.

**Figure 2:**
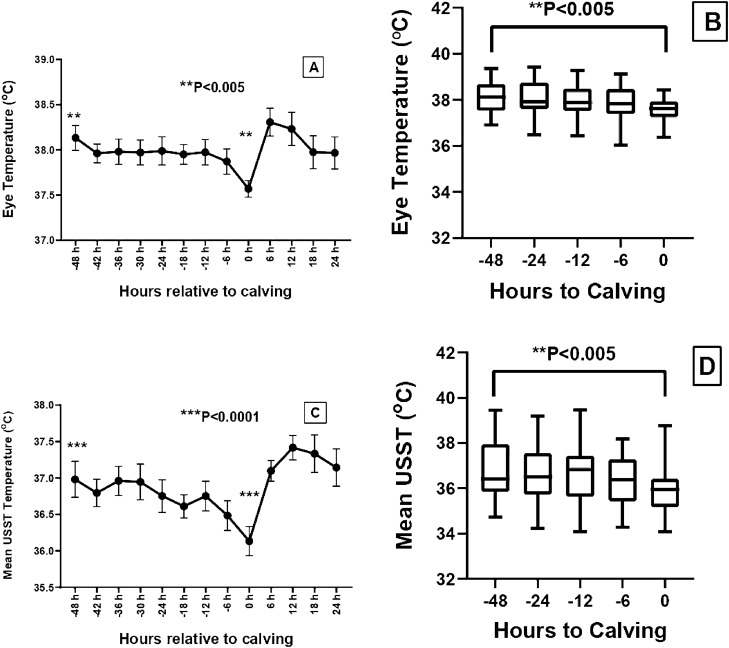
Infrared thermal profile of eye and overall mean of udder skin surface temperature (USST) from 48 h prior to calving to 24 h post calving in buffaloes. (**A**) Eye temperature, (**B**) distribution of Tmax of eye; (**C**) Overall mean of udder skin surface temperature, (**D**) distribution of Tmax of mean udder skin surface. Asterisks (**) and (***) indicate the time points with significant difference in mean surface temperatures at P < 0.005 and P < 0.0001, respectively. Values presented as mean ± SE.

**Figure 3:**
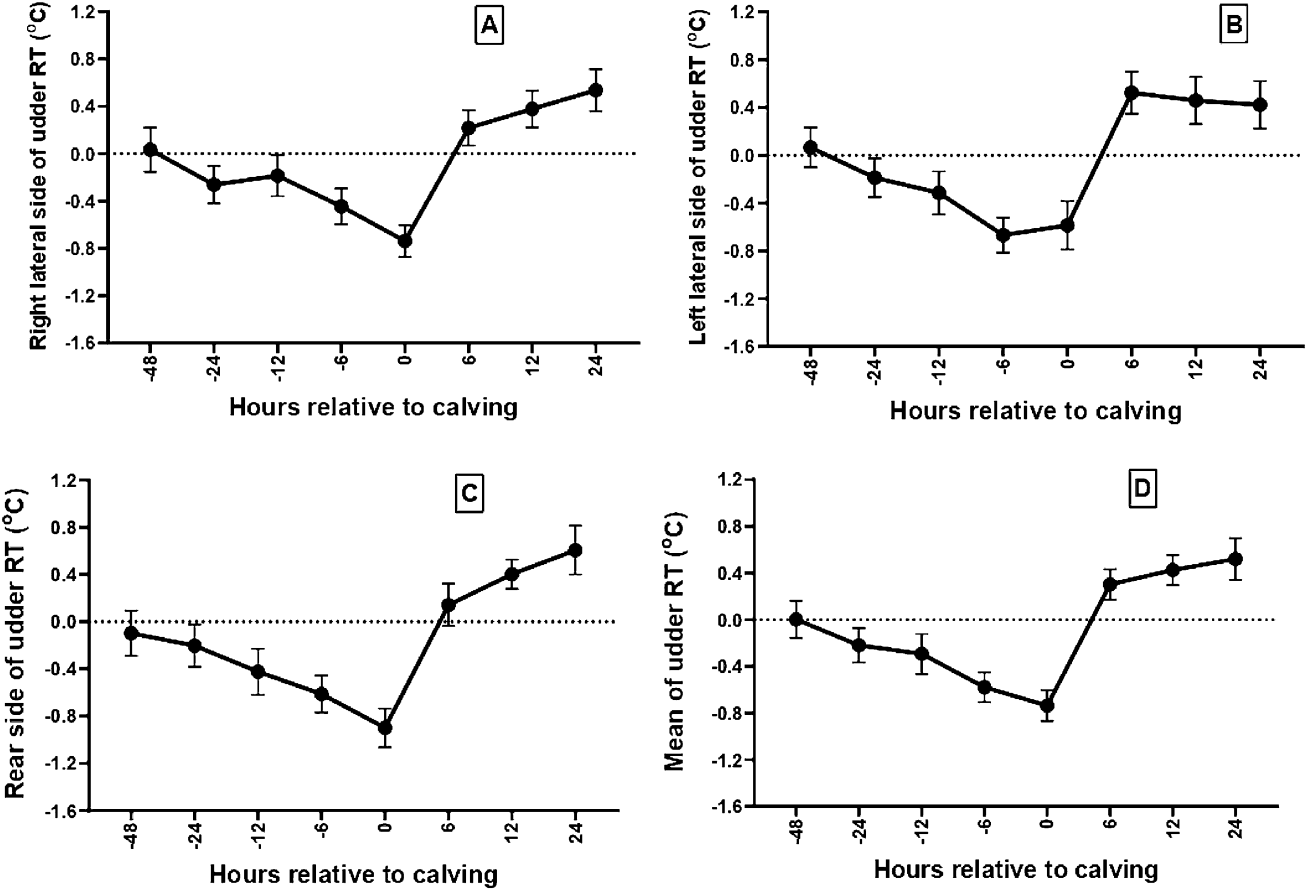
Residual temperatures (RT) of (**A**) right lateral, (**B**) left lateral and (**C**) rear side and (**D**) overall mean RTs of Udder skin surface in buffaloes from 48 h prior to calving to 24 h post calving. Values presented as Mean ± SE.

The mean ± SE of the eye and USST at different time relative to calving (0 h) is presented in Table 1. The mean eye temperature showed a declining trend from 48 h pre-calving till calving. There was a significant reduction in the temperature at the event of calving (37.57 °C) with a ΔT of 0.56 °C in comparison to temperature around 48 h prior to calving (38.13 °C). The rear, left and right lateral USST during the last 48 h pre-calving was significantly different as compared to the temperature at the onset of calving with a ΔT (°C) of 0.94 (P < 0.01), 0.70 (P > 0.05) and 0.91 (P < 0.05) for rear, left and right lateral sides of udder, respectively. However, the thermal profile was comparable from 12 h till the expulsion of calf. The mean USST at 12 h pre-calving was significantly higher than that of 0 h calving with a ΔT of 0.85 °C (P < 0.0001). The mean udder (Fig. 1) and eye temperature (Fig. 2) was significantly (P < 0.005) lower at calving in comparison to 48 h pre-calving. The eye temperature and USST at 12–6 h pre-calving was significantly different from other time points (Fig. 2). The changes in USST during the last 48 h pre-calving (Table 1) and residual temperature of USST followed a similar trend (Fig. 3).

**Table 1:** Estimated marginal means of eye and udder skin surface temperature (USST) at different timepoint interval from 48 h prior calving (-48 h) to calving time (0 h) using linear mixed model of repeated measures of ANOVA and delta (Δ) T (^°^C) with respect to -48 h to 0 h.

Body region	Eye and udder skin surface temperature (mean ± SE) at different time (h) point interval relative to calving (0 h)					ΔT (^°^C) (temperature difference between − 48 h and 0 h calving)
	− 48	− 24	− 12	− 6	0	
Eye	38.13 ± 0.14^a^ (0.194 ± 0.110)	37.99 ± 0.16 (− 0.036 ± 0.103)	37.97 ± 0.16 (− 0.089 ± 0.115)	37.87 ± 0.13 (− 0.193 ± 0.090)	37.57 ± 0.09^b^ (− 0.424 ± 0.157)	0.56
Rear side USST	36.82 ± 0.24^a^ (− 0.099 ± 0.192)	36.66 ± 0.24^a^ (− 0.203 ± 0.179)	36.57 ± 0.29 (− 0.424 ± 0.195)	36.37 ± 0.22 (− 0.613 ± 0.156)	35.88 ± 0.19^b^ (− 0.900 ± 0.166)	0.94
Left lateral USST	37.06 ± 0.24^a^ (0.065 ± 0.167)	36.84 ± 0.22 (− 0.188 ± 0.161)	36.72 ± 0.30 (− 0.315 ± 0.179)	36.48 ± 0.24 (− 0.669 ± 0.147)	36.36 ± 0.23^b^ (− 0.586 ± 0.203)	0.70
Right lateral USST	37.07 ± 0.25^a^ (0.033 ± 0.186)	36.77 ± 0.22^a^ (− 0.263 ± 0.158)	36.96 ± 0.29^a^ (− 0.187 ± 0.174)	36.60 ± 0.25 (− 0.446 ± 0.150)	36.16 ± 0.19^b^ (− 0.738 ± 0.134)	0.91
Mean USST	36.98 ± 0.22^a^ (0.002 ± 0.159)	36.76 ± 0.21^a^ (− 0.220 ± 0.147)	36.75 ± 0.31 (− 0.293 ± 0.172)	36.48 ± 0.24 (− 0.579 ± 0.128)	36.13 ± 0.18^b^ (− 0.736 ± 0.132)	0.85

The values with in parenthesis indicate residual temperature (mean ± SE).

Residual temperatures were calculated using the formula (RT = actual surface temperature – mean surface temperature for the same hour on the previous 3 days).

Values bearing different superscripts between columns differs significantly at P < 0.05.

**Figure 4:**
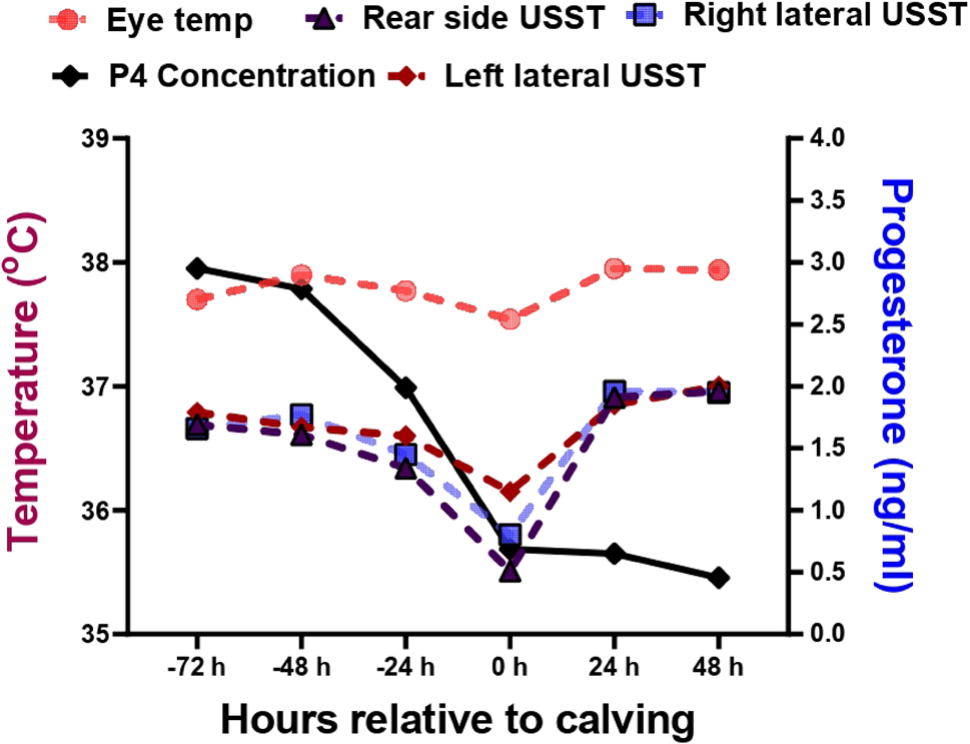
Variations in eye, right lateral, left lateral and rear side of udder skin surface temperature and plasma progesterone (P4) concentration during 72 h prior to calving to 48 h post calving in buffaloes (0 h indicates time of calving).

The P_4_ concentration declined from 2.95 ± 0.16 ng/mL at 72 h pre-calving to 0.69 ± 0.05 ng/mL at calving and remained below 1 ng/mL till 48 h post-calving (Fig. 4). It is apparent from the ROC curve that P_4_ at the concentration of 1.7 ng/mL showed 100% diagnostic sensitivity (95% CI − 83.9 to 100) and specificity 95% (95% CI − 76.4 to 100) − 48 h before calving. This is supported by the positive likelihood ratio of 20 indicating its predictive power. However, we found non-significant correlation between P_4_ concentration with that of eye temperature or USST at any point of calving.

The area under curve (AUC) was used to rank the region of interest (ROI) to forecast calving (Table 2). The AUC values for the eye temperature, rear side USST, right lateral USST and left lateral USST were 0.73, 0.69, 0.69 and 0.64, respectively. The diagnostic sensitivity (Se), specificity (Sp) and threshold (cut-off) values for each ROI are presented in Table 2. The overall mean USST showed a Se and Sp of 57.1% and 75%, respectively at the threshold temperature of 36.77 °C.

**Table 2:** The predictive value of individual region of interest for predicting the calving process in buffalo within 48 h.

Rank	Region of interest	Cut-off value (°C)	AUC*	Sensitivity (%)	Specificity (%)	P value
I	Eye (Body)	38.12	0.730	50.00	89.29	0.003
II	Rear side USST	36.26	0.694	57.14	71.43	0.013
III	Right lateral USST	37.51	0.687	39.29	85.71	0.016
IV	Left lateral USST	37.56	0.639	35.71	78.57	0.074
Overall mean USST		36.77	0.688	57.14	75.00	0.016

*Area under curve (AUC) was determined in the receiver operating characteristic (ROC) analysis.

## Discussion

Reports on the reduction in body temperature from 48 to 24 h pre-calving dates back to 1910^24^. In parallel, it is also well known that the temperature in different regions of interest shows a slight variation with core body temperature. Physiological or pathological events in the udder and environmental factors alter the perfusion pattern of the ROI, which is reflected in the form of altered surface temperature^25^. We chose udder as the primary ROI for monitoring the temperature as its thermal profile is driven by the underlying endocrine changes that orchestrate the calving process. Further, the vast surface area and the caudoventral location of the udder provide additional advantage of recording the temperature with minimum disturbance to the buffalo. The udder was divided into different ROI and the thermal profile was compared with that of eye temperature as it truly represents the core body temperature and is reported to be useful in detecting mastitis^26^. Infrared thermography was the choice of recoding temperature as it is a non-contact method and is of practical value in the dairy cow^6^. Though wallowing is common in the buffalo, the requirement of clean and dirt free surface of the udder for the IRT can be easily achieved under practical settings. It is because of the fact that the advanced pregnant buffaloes are usually restrained from wallowing as per the traditional husbandry practices. In fact, in the present study, advanced–stage pregnant buffaloes were separated from the herd and housed in an individual calving pen 14 days prior to expected date of calving and up to 4 days post-calving. None of the buffalo in the experimental group was allowed for wallowing in water or mud. A significant decline in the temperature of eye and USST 48 h pre-calving is likely to be mediated by the decreasing concentrations of P_4_, which is a thermogenic hormone.

A significant decrease in the eye temperature and USST at 48 h pre-calving in the buffalo is supported by a concomitant decline in the plasma concentration of thermogenic P_4_ hormone. In the present study, eye temperature showed the best thermal window for monitoring changes in the body temperature, which is supported by earlier reports^27,28^. It is reported that orbital region is the best thermal window for evaluating the thermal status and comfort of buffaloes, as the maximum temperature of this region corresponds close to core body temperature (rectal temperature) and least affected by ambient temperature^29,30^. In the prepartum cows and heifers, the body temperature declined significantly (P < 0.05) from 38.9 to 38.6 °C with a ΔT of 0.3 ± 0.5 °C during the last 24 h before parturition^31^.

The temperature at the rear side of the udder surface declined significantly by 0.24 °C (P < 0.001) around calving as compared to 48 h pre-calving. Further, the temperature difference was greater than those of left, right and lateral sides of udder surfaces. These findings are supported by earlier report where caudal region of USST differed and showed higher temperature (0.2–0.9 °C)^32^. An increase in the USST as compared to the core body temperature is due to increased perfusion and greater cutaneous vascularisation of the udder. In contrast to our findings^27,33,34^, an increased temperature difference was recorded in the forequarter area of breed of cow^23,26^ and no significant difference was observed in the USST in Deoni (*Bos indicus*) cows^35^. Similarly, the residual temperatures of both eye and various ROI of udder also followed a similar trend from 48 to 0 h of calving indicating the minimal influence of circadian effect on the USST. A drop in the USST from 12 to 6 h prior to calving might be due to preferential diversion of the circulation to the abdominal muscles and tubular reproductive tract for the expulsion of fetus. An increase in the intramammary pressure is reported to reduce the blood flow to the udder in the goats^36,37^. Interestingly, the USST increased sharply within 6 h post-calving that might be due to the suckling activity of the new born calf.

As the decline in the temperature of eye and USST was significant from 48 to 6 h pre-calving as compared with the temperature at calving, we evaluated the potential in calving prediction in the buffalo. From Table 2, it is evident that the diagnostic sensitivity of eye temperature and USST ranged from 50 to 57% at 48 h pre-calving indicating its low utility in predicting the calving in the buffalo. Any technique for calving prediction should have a diagnostic sensitivity of > 95% as one can afford a maximum false positive rate (1-Sp) of 5%. Further, a calving prediction tool with a minimum diagnostic specificity of 90% is preferred as one can allow a maximum false negative rate of 10%.

It is concluded that the eye temperature and USST showed a significant decline at 48 h pre-calving with a marked decline at 6–12 h. The prepartum decrease in the temperature coincides with a drop in plasma P_4_ concentration. A large-scale study is warranted to enhance the Se and SP and to fix a precise threshold value of USST as a tool for calving prediction.

## Materials and methods

### Ethics approval and consent to participate

The present study was conducted according to the guidelines and approval of the Institutional Animal Ethical Committee of Southern Regional Station of ICAR- National Dairy Research Institute, Bengaluru for the care and use of experimental animals (Approval number: CPCSEA/IAEC/LA/SRS-ICAR-NDRI-2021/No.08). Further, all methods reported in the present study were conducted as per Animal Research: Reporting of In Vivo Experiments (ARRIVE) guidelines. During the entire period of study, none of the experimental buffalo was subjected to either anaesthesia or euthanasia.

### Study location and experimental animals

The current study was conducted between August 2021 and February 2022 in the Murrah buffaloes maintained at the Buffalo Research Station, Venkataramannagudem (Sri Venkateswara Veterinary University, Tirupati, Andhra Pradesh, India). Twenty-eight (n = 28) multiparous pregnant Murrah buffaloes in 3rd to 7th parity were selected. Loose housing system is practiced. The experimental buffaloes were housed in individual calving pens 14 days prior to the expected date of calving. The pregnant buffaloes had free access to green fodder and water while concentrate was given twice a day as per the Indian Council of Agricultural Research recommendations^38^. The buffaloes were apparently healthy and calved normally.

### Measurement of weather parameters and temperature humidity index (THI)

Weather parameters such as rainfall, maximum temperature (Tmax) and minimum temperature (Tmin), Relative humidity (RH), and wind speed were taken from Krishi Vigyan Kendra meteorological station of Horticulture University, which was located in the same premises of the study area. Dry bulb and wet bulb temperature were recorded at the time of thermal image capturing which was done at 6 h interval (05, 11, 17, and 23 h) throughout the experimental period. The ambient temperature and humidity data were fed to the software for analysis while processing and interpreting the thermal image. In the present study, temperature humidity index was calculated as per the formula^39^ which is mentioned below:

$$ {\text{THI }} = \, \left( {{\text{Db }} + {\text{ Wb}}} \right) \, \times \, 0.{72 } + { 4}0.{6} $$

where, Db is dry bulb temperature and Wb is wet bulb temperature.

The influence of calculated THI was correlated with the temperature differences of USST at different time points.

### Blood sampling and hormonal analysis

Blood of ~ 4 mL was collected at aseptically by jugular venipuncture in a vacutainer tube containing 0.5% EDTA (10 mL, BD Vacutainer, K2E (EDTA) 18.0 mg of BD, USA) and centrifuged at 1408.7*g* for 15 min to collect the plasma into two mL sterile screw capped storage vials. Blood sampling was done at 24 h interval from 96 h of expected calving, at the time of calving to 96 h post-calving continuously, but sample was evaluated during the period of − 72 to 48 h post-calving for progesterone estimation. The labelled plasma samples were stored in 2 aliquots at − 20 °C until assay for P_4_ hormone. Plasma P_4_ concentration (ng/mL) was determined by Radio immunoassay kit (RIA Progesterone, Beckman Coulter, Immunotech, Czech Republic: Cat. #IM1188) as per the manufacturer’s guidelines.

### Infrared thermal imaging and analysis

**Figure 5:**
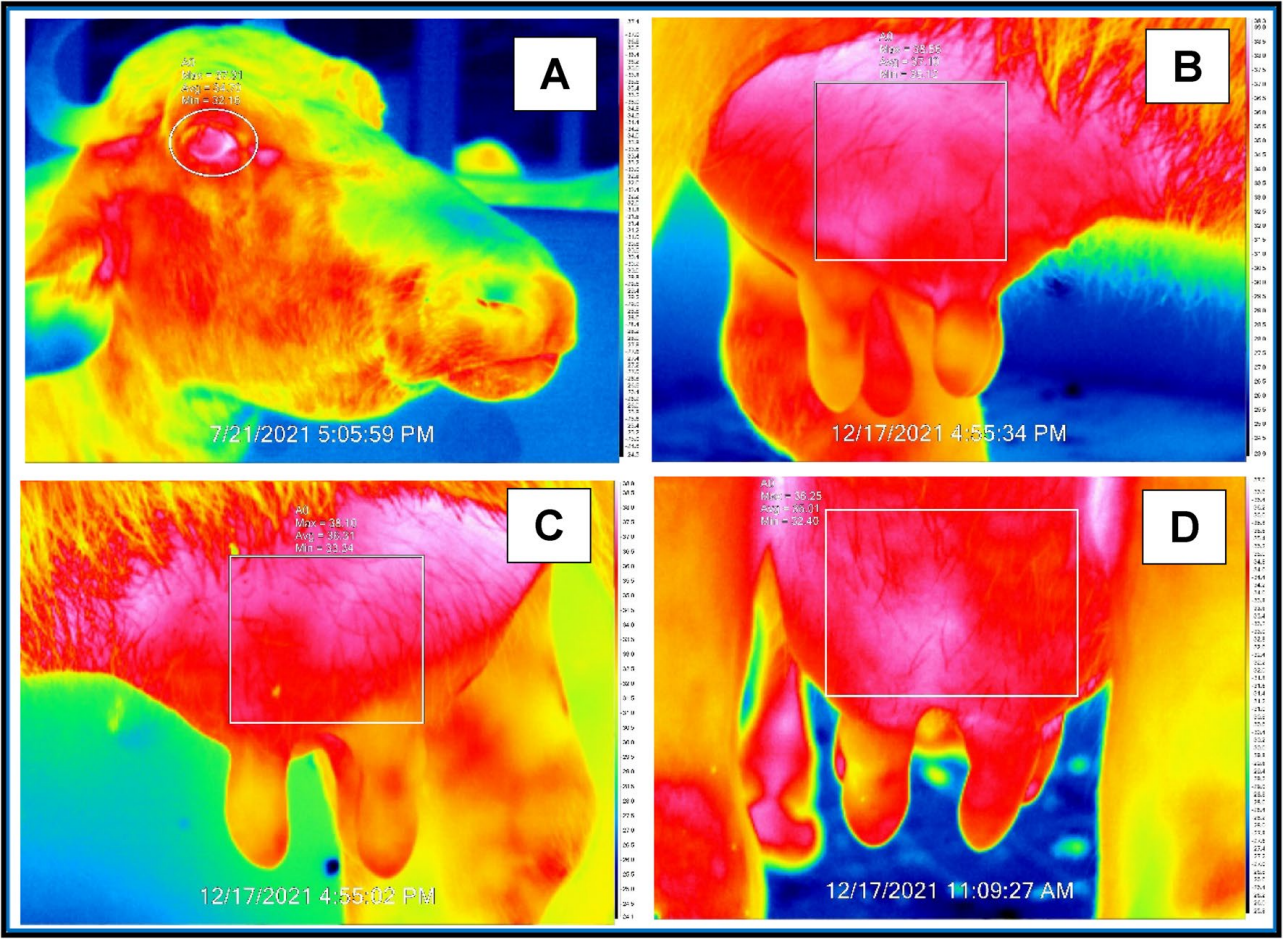
Infrared thermogram of (**A**) eye, (**B**) right lateral, (**C**) left lateral and (**D**) rear side of udder skin surface in buffaloes. The circle and squares in the thermal pictures represent the area used to identify the maximum radiated temperature (23.9–39.4 °C) for each region of interest (ROI).

Thermal images of the eye and udder skin surface were recorded in the experimental buffaloes using a FLUKE Ti32S IRT imaging camera (FLUKE). The IRT was profiled at 6 h interval (05, 11, 17, and 23 h) from 96 h of expected calving till calving and at 24 h post-calving. In the present study, we used a more sensitive camera with thermal sensitivity of ≤ 0.05 °C, a Noise Equivalent Temperature Difference (NETD) of 50 milliKelvins and a pixel resolution of 76,800. Prior to capturing the thermal image at the ROI, the camera was calibrated to ambient temperature. The unit was set to degree Celsius and distance to meters using the in-built software. The values of emissivity and reflected apparent temperature were kept constant for all the images as 0.98 and 20.0 °C, respectively. The recording was done in a confined shed where influence of direct sunlight and wind movements were minimum. The individual buffalo udder surface was wiped off the extraneous dirt and moisture using a clean dry towel 10 min prior to capturing the thermal images. A lateral thermographic image was taken at a distance of 1.0 m from the animal’s head around the eye region (Fig. 5) including the ocular globe, inner canthus, the skin surrounding the ocular cavity and at lacrimal caruncle. Thermographic images of udder were taken at a distance of 1.0 m from the udder and captured from three sides of the udder viz., right lateral, left lateral and rear sides (Fig. 5). A total of 924 ocular and 2772 udder images were taken throughout the experimental period. Images were stored in a memory card and transferred to a laptop for analysis using Fluke connect thermal image analysis software. The IRT images were recorded and processed to find out the ΔT between two time points. Maximum IRT temperatures of ocular and udder regions were used in the analysis, changes in surface temperature were expressed also as residual temperatures. Residual temperatures were calculated using the formula; RT = actual surface temperature (ST) – mean ST for the same hour on the previous 3 days, to eliminate the circadian effects of the data^7,40^. A free hand drawn elliptical shape covering the ocular region and rectangular shape marker were used for the calculation of temperatures of both ocular and udder region thermal images, respectively.

### Statistical analysis

Descriptive statistics and correlation of the data were generated using a Microsoft Excel-2016. Linear mixed model was used by fitting time as fixed effect and buffalo as random effect using SPSS 16.0 (IBM Corporation, Armonk, New York, USA). The results are expressed as mean ± standard error. Keeping the temperature at 0 h (time of calving) as control, the temperature at 48 h pre-calving was used to predict the calving, receiver operative characteristic curve (ROC) analysis was performed using GraphPad Prism version 5.0 (San Diego, CA, USA). The threshold temperature for calving prediction was determined based on Youden’s J index that showed maximum true positive rate (sensitivity) and minimum false positive rate (1-specificity). Significance was set at 95%. Chart was prepared using GraphPad Prism 5.0.

### Supplementary Information

Supplementary Tables.

## Data Availability

The data presented in this study are available from the corresponding author on a reasonable request.

## Abbreviations

ROI: Region of interest
USST: Udder skin surface temperature
P4: Progesterone
THI: Thermal humidity index
RIA: Radio immunoassay
ROC: Receiver operative characteristic curve
