# Supplementary material for: Digital infrared thermal imaging of udder skin surface temperature: a novel non-invasive technology to monitor calving process in Murrah buffalo (Bubalus bubalis)
Source: Sci Rep. 2023 Aug 14;13:13207. doi: 10.1038/s41598-023-40447-4 (PMC10425463; doi:10.1038/s41598-023-40447-4)
Supplement: Supplementary file 1 — Supplementary Tables. [file 41598_2023_40447_MOESM1_ESM.docx]

**Supplementary table 1: Macro and micro weather parameters measured at Buffalo Research Station (study location) during the experimental period.**

| **Macro weather parameters** | | | | | | **Micro weather parameters** | | |
| --- | --- | --- | --- | --- | --- | --- | --- | --- |
| **Month** | **Rainfall**  **(mm)** | **T max.**  **(^o^C)** | **T min.**  **(^o^C)** | **RH**  **(%)** | **Wind Speed**  **(kmph)** | **DB**  **(^o^C)** | **WB**  **(^o^C)** | **THI** |
| Aug-21 | 7.00 | 32.42 | 25.75 | 92.52 | 1.17 | 28.64 | 26.18 | 80.07 |
| Sep-21 | 7.40 | 33.10 | 26.13 | 91.42 | 1.17 | 28.36 | 26.24 | 79.91 |
| Oct-21 | 1.09 | 34.41 | 25.39 | 86.47 | 1.06 | 28.77 | 26.89 | 80.67 |
| Nov-21 | 3.62 | 30.43 | 23.25 | 88.64 | 1.31 | 26.18 | 24.06 | 76.77 |
| Dec-21 | 0.02 | 30.89 | 19.71 | 79.28 | 1.15 | 23.25 | 20.36 | 72.00 |
| Jan-22 | 0.73 | 29.83 | 19.14 | 80.42 | 1.35 | 23.95 | 21.06 | 73.01 |
| Feb-22 | 0.00 | 30.76 | 19.04 | 76.53 | 1.22 | 24.50 | 21.64 | 73.82 |

**Supplementary table 2.1: Descriptive statistics of ocular region (eye) temperature at each crucial time point before 96 hours calving and up to 24 hours post calving in buffalo measured by infrared thermal imaging.**

| **S.No.** | **Descriptive measures** | **Hours prior, during (0 h) and post calving** | | | | | | | | | | | | | | | | | | | | |
| --- | --- | --- | --- | --- | --- | --- | --- | --- | --- | --- | --- | --- | --- | --- | --- | --- | --- | --- | --- | --- | --- | --- |
|  |  | **-96** | **-90** | **-84** | **-78** | **-72** | **-66** | **-60** | **-54** | **-48** | **-42** | **-36** | **-30** | **-24** | **-18** | **-12** | **-6** | **0** | **6** | **12** | **18** | **24** |
| 1 | Mean | 38.08 | 38.24 | 38.14 | 38.05 | 37.86 | 38.08 | 38.07 | 38.17 | 38.13 | 37.96 | 37.98 | 37.97 | 37.99 | 37.95 | 37.97 | 37.87 | 37.57 | 38.31 | 38.23 | 37.97 | 37.97 |
| 2 | SE | 0.14 | 0.18 | 0.16 | 0.12 | 0.14 | 0.14 | 0.15 | 0.16 | 0.14 | 0.10 | 0.14 | 0.14 | 0.16 | 0.11 | 0.14 | 0.14 | 0.09 | 0.15 | 0.18 | 0.18 | 0.18 |
| 3 | SD | 0.73 | 0.96 | 0.85 | 0.66 | 0.73 | 0.75 | 0.80 | 0.83 | 0.73 | 0.55 | 0.74 | 0.73 | 0.83 | 0.58 | 0.75 | 0.75 | 0.48 | 0.81 | 0.96 | 0.97 | 0.94 |
| 4 | Median | 38.07 | 38.11 | 37.89 | 37.96 | 37.82 | 37.87 | 37.98 | 38.16 | 38.12 | 38.01 | 38.01 | 37.89 | 37.93 | 38.01 | 37.89 | 37.84 | 37.63 | 38.38 | 38.34 | 37.82 | 37.91 |
| 5 | Range | 3.13 | 3.10 | 3.17 | 2.48 | 2.70 | 2.65 | 3.21 | 3.36 | 2.45 | 2.40 | 3.12 | 3.15 | 2.94 | 2.28 | 2.84 | 3.09 | 2.06 | 3.20 | 3.65 | 3.57 | 3.25 |
| 6 | Minimum | 36.04 | 36.71 | 36.30 | 37.14 | 36.67 | 36.95 | 36.52 | 36.42 | 36.91 | 36.95 | 36.16 | 36.51 | 36.49 | 36.85 | 36.44 | 36.04 | 36.37 | 36.57 | 36.12 | 36.07 | 36.39 |
| 7 | Maximum | 39.17 | 39.81 | 39.47 | 39.62 | 39.37 | 39.60 | 39.73 | 39.78 | 39.36 | 39.35 | 39.28 | 39.66 | 39.43 | 39.13 | 39.28 | 39.13 | 38.43 | 39.77 | 39.77 | 39.64 | 39.64 |
| 8 | Count | 28 | 28 | 28 | 28 | 28 | 28 | 28 | 28 | 28 | 28 | 28 | 28 | 28 | 28 | 28 | 28 | 28 | 28 | 28 | 28 | 28 |

**Supplementary table 2.2: Descriptive statistics of right-side udder skin surface temperature at each crucial time point before 96 hours calving and up to 24 hours post calving in buffalo measured by infrared thermal imaging.**

| **S.No.** | **Descriptive measures** | **Hours prior, during (0 h) and post calving** | | | | | | | | | | | | | | | | | | | | |
| --- | --- | --- | --- | --- | --- | --- | --- | --- | --- | --- | --- | --- | --- | --- | --- | --- | --- | --- | --- | --- | --- | --- |
|  |  | **-96** | **-90** | **-84** | **-78** | **-72** | **-66** | **-60** | **-54** | **-48** | **-42** | **-36** | **-30** | **-24** | **-18** | **-12** | **-6** | **0** | **6** | **12** | **18** | **24** |
| 1 | Mean | 37.14 | 37.09 | 37.01 | 36.96 | 36.87 | 37.02 | 37.34 | 37.24 | 37.07 | 36.87 | 37.1 | 36.93 | 36.77 | 36.79 | 36.96 | 36.6 | 36.16 | 37.11 | 37.51 | 37.42 | 37.2 |
| 2 | SE | 0.25 | 0.27 | 0.23 | 0.28 | 0.25 | 0.23 | 0.21 | 0.26 | 0.27 | 0.23 | 0.2 | 0.26 | 0.23 | 0.18 | 0.19 | 0.22 | 0.2 | 0.14 | 0.21 | 0.26 | 0.26 |
| 3 | SD | 1.33 | 1.42 | 1.22 | 1.49 | 1.32 | 1.21 | 1.13 | 1.4 | 1.45 | 1.23 | 1.04 | 1.36 | 1.23 | 0.93 | 1 | 1.18 | 1.07 | 0.73 | 1.09 | 1.38 | 1.39 |
| 4 | Median | 37.22 | 37.12 | 36.78 | 36.76 | 36.94 | 37.03 | 37.28 | 37.05 | 37.13 | 36.84 | 37.06 | 37.06 | 36.82 | 36.91 | 37.06 | 36.82 | 35.78 | 37.01 | 37.58 | 37.36 | 37.27 |
| 5 | Range | 4.54 | 5.62 | 4.59 | 5.01 | 5.69 | 4.33 | 3.65 | 4.86 | 6.14 | 5.49 | 4.38 | 5.82 | 4.46 | 3.74 | 4.02 | 4.29 | 3.95 | 2.92 | 4.22 | 5.41 | 5.44 |
| 6 | Minimum | 34.85 | 34.13 | 34.81 | 34.8 | 34.42 | 34.72 | 35.71 | 35.1 | 34.15 | 34.25 | 34.67 | 34.15 | 34.66 | 34.63 | 35.12 | 34.19 | 34.24 | 35.61 | 35.31 | 34.36 | 35.02 |
| 7 | Maximum | 39.39 | 39.75 | 39.4 | 39.81 | 40.11 | 39.05 | 39.36 | 39.96 | 40.29 | 39.74 | 39.05 | 39.97 | 39.12 | 38.37 | 39.14 | 38.48 | 38.19 | 38.53 | 39.53 | 39.77 | 40.46 |
| 8 | Count | 28 | 28 | 28 | 28 | 28 | 28 | 28 | 28 | 28 | 28 | 28 | 28 | 28 | 28 | 28 | 28 | 28 | 28 | 28 | 28 | 28 |

**Supplementary table 2.3: Descriptive statistics of left side udder skin surface temperature at each crucial time point before 96 hours calving and up to 24 hours post calving in buffalo measured by infrared thermal imaging.**

| **S.No.** | **Descriptive measures** | **Hours prior, during (0 h) and post calving** | | | | | | | | | | | | | | | | | | | | |
| --- | --- | --- | --- | --- | --- | --- | --- | --- | --- | --- | --- | --- | --- | --- | --- | --- | --- | --- | --- | --- | --- | --- |
|  |  | **-96** | **-90** | **-84** | **-78** | **-72** | **-66** | **-60** | **-54** | **-48** | **-42** | **-36** | **-30** | **-24** | **-18** | **-12** | **-6** | **0** | **6** | **12** | **18** | **24** |
| 1 | Mean | 37.08 | 37.24 | 36.91 | 37.01 | 36.94 | 36.98 | 37.24 | 37.46 | 37.06 | 36.76 | 36.96 | 36.98 | 36.84 | 36.58 | 36.72 | 36.48 | 36.36 | 37.3 | 37.43 | 37.37 | 37.18 |
| 2 | SE | 0.21 | 0.28 | 0.22 | 0.26 | 0.26 | 0.23 | 0.23 | 0.27 | 0.26 | 0.21 | 0.21 | 0.25 | 0.23 | 0.19 | 0.22 | 0.22 | 0.25 | 0.19 | 0.19 | 0.27 | 0.27 |
| 3 | SD | 1.09 | 1.49 | 1.17 | 1.38 | 1.37 | 1.22 | 1.23 | 1.44 | 1.4 | 1.1 | 1.13 | 1.31 | 1.2 | 1.02 | 1.15 | 1.18 | 1.33 | 0.98 | 1.01 | 1.44 | 1.43 |
| 4 | Median | 37.17 | 37.22 | 36.72 | 36.67 | 36.82 | 36.96 | 37.19 | 37.99 | 37.16 | 36.93 | 36.9 | 37.18 | 36.77 | 36.74 | 36.66 | 36.36 | 36.17 | 37.51 | 37.63 | 37.14 | 37.06 |
| 5 | Range | 3.57 | 5.83 | 4.33 | 5.1 | 5.31 | 3.76 | 4.56 | 5.54 | 5.12 | 4.14 | 4.68 | 5.4 | 5.15 | 4.12 | 4.41 | 3.55 | 4.51 | 3.81 | 3.69 | 5.13 | 5.77 |
| 6 | Minimum | 35.43 | 34.14 | 35.27 | 35.07 | 34.74 | 35.05 | 35.23 | 34.14 | 34.48 | 34.15 | 34.81 | 34.05 | 34.43 | 34.34 | 35 | 34.6 | 34.19 | 35.36 | 35.19 | 34.95 | 34.15 |
| 7 | Maximum | 39 | 39.97 | 39.6 | 40.17 | 40.05 | 38.81 | 39.79 | 39.68 | 39.6 | 38.29 | 39.49 | 39.45 | 39.58 | 38.46 | 39.41 | 38.15 | 38.7 | 39.17 | 38.88 | 40.08 | 39.92 |
| 8 | Count | 28 | 28 | 28 | 28 | 28 | 28 | 28 | 28 | 28 | 28 | 28 | 28 | 28 | 28 | 28 | 28 | 28 | 28 | 28 | 28 | 28 |

**Supplementary table 2.4: Descriptive statistics of rear side udder skin surface temperature at each crucial time point before 96 hours calving and up to 24 hours post calving in buffalo measured by infrared thermal imaging.**

| **S.No.** | **Descriptive measures** | **Hours prior, during (0 h) and post calving** | | | | | | | | | | | | | | | | | | | | |
| --- | --- | --- | --- | --- | --- | --- | --- | --- | --- | --- | --- | --- | --- | --- | --- | --- | --- | --- | --- | --- | --- | --- |
|  |  | **-96** | **-90** | **-84** | **-78** | **-72** | **-66** | **-60** | **-54** | **-48** | **-42** | **-36** | **-30** | **-24** | **-18** | **-12** | **-6** | **0** | **6** | **12** | **18** | **24** |
| 1 | Mean | 36.94 | 37.1 | 36.85 | 36.85 | 36.83 | 36.97 | 37.22 | 37.14 | 36.8 | 36.7 | 36.95 | 36.89 | 36.65 | 36.45 | 36.58 | 36.35 | 35.86 | 36.85 | 37.32 | 37.16 | 37.04 |
| 2 | SE | 0.2 | 0.2 | 0.27 | 0.29 | 0.23 | 0.18 | 0.25 | 0.28 | 0.26 | 0.24 | 0.2 | 0.26 | 0.24 | 0.15 | 0.24 | 0.21 | 0.21 | 0.19 | 0.18 | 0.28 | 0.27 |
| 3 | SD | 1.06 | 1.06 | 1.43 | 1.54 | 1.22 | 0.94 | 1.3 | 1.46 | 1.36 | 1.25 | 1.08 | 1.36 | 1.27 | 0.8 | 1.28 | 1.09 | 1.1 | 1 | 0.97 | 1.47 | 1.41 |
| 4 | Median | 36.85 | 37.24 | 36.74 | 36.4 | 36.65 | 36.9 | 36.95 | 37.12 | 36.42 | 36.58 | 36.94 | 37.07 | 36.51 | 36.51 | 36.83 | 36.38 | 35.96 | 36.71 | 37.6 | 36.91 | 37.29 |
| 5 | Range | 3.87 | 4.77 | 5.06 | 5.85 | 5.72 | 4.07 | 4.83 | 5.05 | 4.71 | 4.66 | 5.2 | 5.38 | 4.96 | 3.22 | 5.37 | 3.89 | 4.7 | 4.21 | 3.24 | 5.19 | 5.63 |
| 6 | Minimum | 35.1 | 34.55 | 34.89 | 34.16 | 34.63 | 34.83 | 34.47 | 34.67 | 34.74 | 34.18 | 34.25 | 34.41 | 34.23 | 34.33 | 34.09 | 34.29 | 34.08 | 35.07 | 35.86 | 34.88 | 34.8 |
| 7 | Maximum | 38.97 | 39.32 | 39.95 | 40.01 | 40.35 | 38.9 | 39.3 | 39.72 | 39.45 | 38.84 | 39.45 | 39.79 | 39.19 | 37.55 | 39.46 | 38.18 | 38.78 | 39.28 | 39.1 | 40.07 | 40.43 |
| 8 | Count | 28 | 28 | 28 | 28 | 28 | 28 | 28 | 28 | 28 | 28 | 28 | 28 | 28 | 28 | 28 | 28 | 28 | 28 | 28 | 28 | 28 |

**Supplementary table 2.5:** **Descriptive statistics of overall mean of udder skin surface temperature at each crucial time point before 96 hours calving and up to 24 hours post calving in buffalo measured by infrared thermal imaging.**

| **S.No.** | **Descriptive measures** | **Hours prior, during (0 h) and post calving** | | | | | | | | | | | | | | | | | | | | |
| --- | --- | --- | --- | --- | --- | --- | --- | --- | --- | --- | --- | --- | --- | --- | --- | --- | --- | --- | --- | --- | --- | --- |
|  |  | **-96** | **-90** | **-84** | **-78** | **-72** | **-66** | **-60** | **-54** | **-48** | **-42** | **-36** | **-30** | **-24** | **-18** | **-12** | **-6** | **0** | **6** | **12** | **18** | **24** |
| 1 | Mean | 37.07 | 37.16 | 36.91 | 36.94 | 36.87 | 36.98 | 37.26 | 37.3 | 36.98 | 36.79 | 36.96 | 36.95 | 36.75 | 36.61 | 36.75 | 36.48 | 36.13 | 37.1 | 37.42 | 37.33 | 37.14 |
| 2 | SE | 0.21 | 0.24 | 0.23 | 0.27 | 0.23 | 0.2 | 0.22 | 0.25 | 0.25 | 0.19 | 0.2 | 0.24 | 0.22 | 0.16 | 0.2 | 0.2 | 0.2 | 0.14 | 0.17 | 0.26 | 0.26 |
| 3 | SD | 1.1 | 1.28 | 1.2 | 1.41 | 1.24 | 1.05 | 1.18 | 1.33 | 1.3 | 0.99 | 1.06 | 1.29 | 1.18 | 0.84 | 1.07 | 1.07 | 1.05 | 0.75 | 0.89 | 1.37 | 1.35 |
| 4 | Median | 37.04 | 37.27 | 36.56 | 36.54 | 36.79 | 37.08 | 37 | 37.38 | 36.83 | 36.54 | 36.79 | 37.16 | 36.64 | 36.63 | 36.61 | 36.42 | 36.02 | 37.12 | 37.39 | 37.1 | 37.07 |
| 5 | Range | 3.58 | 5.27 | 4.63 | 4.88 | 5.05 | 3.68 | 4.01 | 4.63 | 5.02 | 3.75 | 4.92 | 5.01 | 4.43 | 3.34 | 4.54 | 3.91 | 3.66 | 2.71 | 3.15 | 4.94 | 5.41 |
| 6 | Minimum | 35.34 | 34.27 | 34.96 | 35.12 | 35.12 | 35.2 | 35.47 | 35.12 | 34.73 | 35.05 | 34.41 | 34.58 | 34.77 | 34.59 | 34.8 | 34.36 | 34.44 | 35.62 | 35.68 | 34.84 | 34.86 |
| 7 | Maximum | 38.92 | 39.54 | 39.59 | 40 | 40.17 | 38.88 | 39.48 | 39.75 | 39.75 | 38.8 | 39.33 | 39.59 | 39.2 | 37.93 | 39.34 | 38.27 | 38.1 | 38.33 | 38.83 | 39.78 | 40.27 |
| 8 | Count | 28 | 28 | 28 | 28 | 28 | 28 | 28 | 28 | 28 | 28 | 28 | 28 | 28 | 28 | 28 | 28 | 28 | 28 | 28 | 28 | 28 |
